# Items, Selections and Remarks

**Published:** 1870-02

**Authors:** W. W. Miner


### Items, Selections and Remarks.

BY W. W. MINER, A. B.

---

At a late meeting of the N. Y. Medical Association, Dr. Knapp stated that in a letter from Prof. G. Simon, of Heidelberg, the writer said that in August last, he had made *successful extirpation of the kidney.* The patient in an operation for ovarian dropsy had had both ovaries and a portion of the uterus removed, and it was afterwards found that one of the ureters had been severed so that the removal of the corresponding kidney was determined upon. The operation was not easy but was accomplished without accident. Some fever was experienced for two weeks, but at the end of six weeks the patient entirely recovered. Dr. Knapp thought the operation of interest, not only as being the newest, but also one of the most remarkable of surgical operations.——Mr. Herard in his experience with the vaccination of 493 new-born infants, has been able to detect no difference between the effects of virus from the cow and that from man.——Drs. Elmer, [illegible]usser and Porter, of Philadelphia, employ the sponge dressing, as a means of applying pressure, with success, in cases of chronic synovitis.——Dr. E. Fitzau, of Germany, relates the case of a physician, 57 years of age, who swallowed 40 grammes of chloroform. Vomiting soon ensued, he then fell into a state of profound anæsthesia. A stomach pump was used, and a flexible tube introduced into the trachea, and at the end of sixteen hours sensibility returned, but acute gastritis terminated the life of the patient 24½ hours after the taking of the dose.——Langenbeck, of Hanover, has recently removed an inverted uterus. The patient removed the ligature on the tenth day. The operation was a success. One of our western surgeons has performed, involuntarily, the same operation with complete success. He supposed that he was removing a uterine polypus. Another case of inversion has narrowly escaped, recently, the operation for the removal of a uterine tumor. Dr. Atlee, of Philadelphia, states that uterine fibroids may, at times, be removed by small doses of muriate of ammonia. This practice, will not, of course, receive the endorsement of the profession, but it has, at least, one merit; a female adopting it may escape the loss of her uterus from the blunders of surgeons, who will, it seems, mistake cases of inversion for tumors, and forthwith institute summary ablation.—*Richmond and Louisville Medical Journal.*

The Bishop of Exeter attained the age of ninety-one years, notwithstanding his life was a most active and stormy one. He is said to have spent from twenty-five to thirty thousand pounds in law suits, and he has written books in so great number that the titles of these alone, occupy thirty pages of the British Museum Catalogue.——A case of feigned sex is reported in the *British Medical Journal.* The deception was not discovered until the death of the individual, when it was found that a person, who, for fifty years, had passed herself off as a man, and who had actually been twice married, was, in reality, of the female sex.—*Reporter.*——Alexis St. Martin, whose case has furnished such remarkable opportunities for observing the functions of digestion, is said to be still alive and well, and lives in Cavendish, Vt.——One diploma vender is now located in jail in Port Clinton, O.

——A "Doctor" in Philadelphia has just been fined $300 and sentenced to one year's imprisonment for publishing obscene books.——Two hundred and fifty cases of relapsing fever have occurred in New York city, and the *Record* says that nine attendants and several of the house physicians in Bellevue Hospital have contracted the disease.——A Dispensary for diseases of the throat and chest has been established at 1354 Broadway, of which Dr. Ruppaner is the attending physician.——Prof. Echeverria, of University Medical College, New York, has instituted a Clinic in Mental Diseases, at the Epileptic and Paralytic Hospital on Blackwell's Island.

The Metropolitan Board of Health has given notice that the same reports to the board are required, respecting cases of relapsing fever as concerning other diseases of a contagious or infectious nature. Dr. Harris says that most of the cases which have thus far occurred, have been among charity patients, and that not unfrequently this affection has not been distinguished from intermittent or typhoid fever.——The Government of India is making efforts to obtain official reports of cases of poisoning from venomous reptiles, and the method employed in the treatment of the same.——The German Hospital, of New York, has received from Germany an unexpected gift of $50,000 in "five-twenties." The giver was the Baron Von Diergardt, a young man, who had just inherrited a large fortune, and begins by making use of it in this manner. He has never been in the United States, and was no doubt, prompted to this great act of charity, by finding a large quantity of U. S. Bonds among his inheritances. The Hospital which is at the corner of 4th Avenue and 77th Street, New York, will be greatly benefitted by this acquisition.—*Reporter.*——Certain male students in the Bellevue Hospital clinic, have followed the example of their Philladelphia fellow-students in giving expression of disapproval of the attendance of women students at the clinic, and in even more severe and impolite terms than were those at the Pennsylvania Hospital.

Profs. T. G. Thomas and Isaac E. Taylor, also Drs. Emmet and Stephen Rogers of New York City, have been elected Corresponding Members of the Obsterical Society of Berlin.—*N. Y. Medical Journal.*——Dr. J. W. Carnochan has been appointed Health Officer of the Port of New York.——Twenty-eight students received the degree of doctor in medicine, from Albany Medical College, on the twenty-third of December last.——The distinguished specialist Ricord, has received a gratifying mark of imperial favor. Like Mr. Nelaton, he has been made Senator. He has also lately received from the Emperor in connection with a cordial letter thanking him for his attention during his recent illness, a gold snuff box ornamented with diamonds valued at 10,000 francs. The messenger who carried the present, took back the answer that the autograph letter would have been prized just as much without the elegantly jewelled envelope.—*Med. Times and Gazette.*——A class of one hundred and forty-one graduates received their degrees from Bellevue Medical College, at the Academy of Music, Feb. 25th.——The Senate will rescind the charter of the Medical Society of the District of Columbia as being behind the spirit of the age.

German Pharmacists publish analyses of patent medicines as the best mode of warfare against this increasing evil. Of those of real medical value they publish the proper value in money which is invariably far below the amount asked.—*Pharmacist.*——Minute examinations of liquids found enclosed in crystals of quartz, topaz, etc., by Geissler and Vogelsang, have shown that what is popularly supposed to be water, and by scientists to be hydro-carbons, is really carbonic acid in a liquid state and not unfrequently mingled with water.——A new kind of gunpowder has been prepared by M. Bruyere, by mixing 54 parts of picrate of ammonia with 46 parts of nitrate of potassa. The mixture produces, on burning, 10 atoms of carbonic acid, 6 of nitrogen, 6 of hydrogen, and as a solid residue, 2 of carbonate of potassa. This powder hardly emits any smoke and what little is emitted is devoid of smell. The residue left after combustion does not affect metals, since it is only carbonate of potassa.—*American Exchange and Review.*——From researches made by M. Marie Davey, it is determined that the calorific influence of the moon upon the earth, compared with that of the sun, is as 1 to 12,000,000.

The item in the last issue of the JOURNAL, stating that a Dr. Chas. Von Graefe, *the distinguished Oculist* was about to visit this country, was taken from the public press. We are informed that Prof. Albrecht Von Graefe has no intention of visiting the United States, and this ruse is probably only one of the forms of quack advertising.

The Medical Society of the state of West Virginia, has recommended to the Legislature the appointment of a State Geologist.——"The Ophthalmological Society of Philadelphia" was organized at a meeting held Feb. 8th, in the Hall of the Pennsylvania Hospital. Dr. I. Hays was elected president, and Dr. W. F. Norris, secretary of the society.——The faculty of the University of Edinburgh has made arrangements for the education of female students of medicine at that institution.——At the time of the death of Prof. Dunglison, there had been issued of his various works, 160,000 volumes. So says Prof. Gross in his memoir of the deceased.—*Pacific Medical Journal.*——A Chicago physician who had been engaged in real estate speculation, is said to have prescribed for a female patient, a drachm of quinine, to be taken—one quarter down—balance in one, two and three years.——The American Publishers Circular announces that D. Appleton & Co., are to publish a work on Chemical Analysis of the Urine, by Austin Flint. M. D., and J. B. Lippincott & Co., a Hand-book of Medical Microscopy, by J. S. Richardson, M. D., also Barnes' Lectures on Obstetric Operations.——The Sydenham Society is about to publish Niemeyer's Lectures on Phthisis, Wunderlich's Treatise on Temperature in Disease, and Strichler's Manual of Human and Comparative Histology.

